# Neck Management in cT1N0 Tongue Squamous Cell Carcinoma as Determined by Sonographic Depth of Invasion

**DOI:** 10.3389/fonc.2021.786258

**Published:** 2022-01-24

**Authors:** Yao Wu, Xu Zhang, Liyuan Dai, Qigen Fang, Wei Du

**Affiliations:** Department of Head Neck and Thyroid, Affiliated Cancer Hospital of Zhengzhou University, Henan Cancer Hospital, Zhengzhou, China

**Keywords:** depth of invasion, tongue squamous cell carcinoma, head and neck squamous cell carcinoma, observation, elective neck dissection

## Abstract

**Objectives:**

To compare the oncologic outcomes in patients with cT1N0 tongue squamous cell carcinoma (SCC) who underwent different neck management strategies stratified by sonographic depth of invasion (DOI).

**Methods:**

The included patients were retrospectively enrolled, and divided into two groups: observation (OBS) and elective neck dissection (END). The regional control (RC) and disease-specific survival (DSS) rates were compared and stratified by sonographic DOI.

**Results:**

The mean sonographic and pathologic DOIs were 3.8 and 3.7 mm, respectively; the two DOIs were significantly correlated (Spearman correlation coefficient 0.974. p <0.001). In patients with sonographic DOI <4.0 mm, the 5-year RC rates were 73 and 89% in the OBS and END groups, respectively, and were not significantly different. However, in patients with sonographic DOI ≥4.0 mm, the 5-year RC rate was significantly different between the OBS (57%) and END (80%) groups (p = 0.031). In patients with sonographic DOI <4.0 mm, the 5-year DSS rates were 79 and 89% in OBS and END groups, respectively, and were not significantly different. However, in patients with sonographic DOI ≥4.0 mm, the 5-year DSS rate was significantly different between the OBS (67%) and END (86%) groups (p = 0.033).

**Conclusions:**

Sonographic DOI was notably correlated with pathologic DOI. Moreover, there was a significant survival difference between the OBS and END groups in cT1N0 tongue SCC patients with sonographic DOI ≥4.0 mm but not in those with sonographic DOI <4.0 mm. Our study provides a useful method to aid decision-making in the clinical setting for this patient group.

## Introduction

Surgical excision is the preferred method for managing squamous cell carcinoma (SCC) of the tongue, which is the most common oral malignancy (1). Neck dissection is usually included in the initial treatment of cT3–T4 disease; however, the optimal neck management in cases of cT1N0 tongue SCC is still controversial owing to the wide range of the occult metastasis rate (2). Observation (OBS) and elective neck dissection (END) are two potential approaches for management. Vandenbrouck et al. (3), Fakih et al. (4), and Yuen et al. (5) reported that a comparison of oncologic outcomes between patients undergoing OBS and those indicated for END revealed a similar disease-specific survival (DSS) in both groups. However, some high-quality studies also showed that END could reduce the frequency of regional nodal recurrence and improve DSS in patients with cT1-2N0 oral SCC (6–8). To achieve successful outcomes in such cases, reliable predictors indicating cervical lymph node metastasis, which can be assessed preoperatively, must be identified.

Factors contributing to lymph node metastasis include tumor size, tumor differentiation grade, perineural invasion (PNI), and lymphovascular invasion (LVI) (9–11). Caponio et al. (12) reported that PNI occurred in 40.5% of 200 patients with tongue SCC, and PNI was associated with a higher tendency of lymph node metastasis and a worse disease prognosis. However, the depth of invasion (DOI) is considered the best predictor of occult lymph node metastasis (13). Studies have suggested that neck dissection should be performed if the DOI exceeds 4 mm (14–16). However, in such studies, the DOI was measured postoperatively based on hematoxylin and eosin staining results; this is known as pathologic DOI, which provides little benefit in preoperative decision-making.

Intraoral ultrasound, CT, and MRI are used to evaluate clinical DOI (17, 18). Takamura et al. (17) reported that compared to pathologic DOI, clinical DOI derived by ultrasound was overestimated by an average of 0.2 mm, while CT and MRI-based radiological DOIs were overestimated by an average of 2–3 mm. These findings, combined with the reports of Klein et al. (19) and Marchi et al. (20), highlight the accuracy of ultrasound in determining the clinical DOI. However, to our knowledge, no study has analyzed whether sonographic DOI can be used to guide neck management in cT1N0 tongue SCC. Therefore, this study aimed to compare the oncologic outcomes in patients that underwent different neck management strategies stratified by sonographic DOI.

## Patients and Methods

### Ethical Considerations

This study was approved by the Institutional Research Committee of our hospital, and all the participants provided informed consent. All procedures involving human participants were conducted according to the ethical standards of the Institutional and National Research Committees and the 1964 Helsinki Declaration and its later amendments or comparable ethical standards.

### Patient Selection

We retrospectively reviewed the medical records of patients that underwent surgical treatment for primary tongue SCC between January 2015 and January 2021. The following were the criteria for study enrollment: a disease stage of cT1N0 according to the 8th AJCC classification system and the availability of follow-up data. Patients with a history of any other malignancy were excluded. Information on demography, treatment, pathology, and follow-up was extracted and analyzed.

### Important Definitions of Variables

A cT1 tumor was defined as a tumor with a maximum diameter of 2 cm and a maximum clinical DOI of 5 mm based on imaging examination. A cN0 neck referred to a neck with no clinically enlarged lymph nodes on palpation and imaging. PNI was considered present if tumor cells were identified within the perineural space and/or nerve bundle. LVI was considered present if tumor cells were noted within the lymphovascular channels (21, 22).

### Evaluation of Clinical DOI

**Figure 1:**
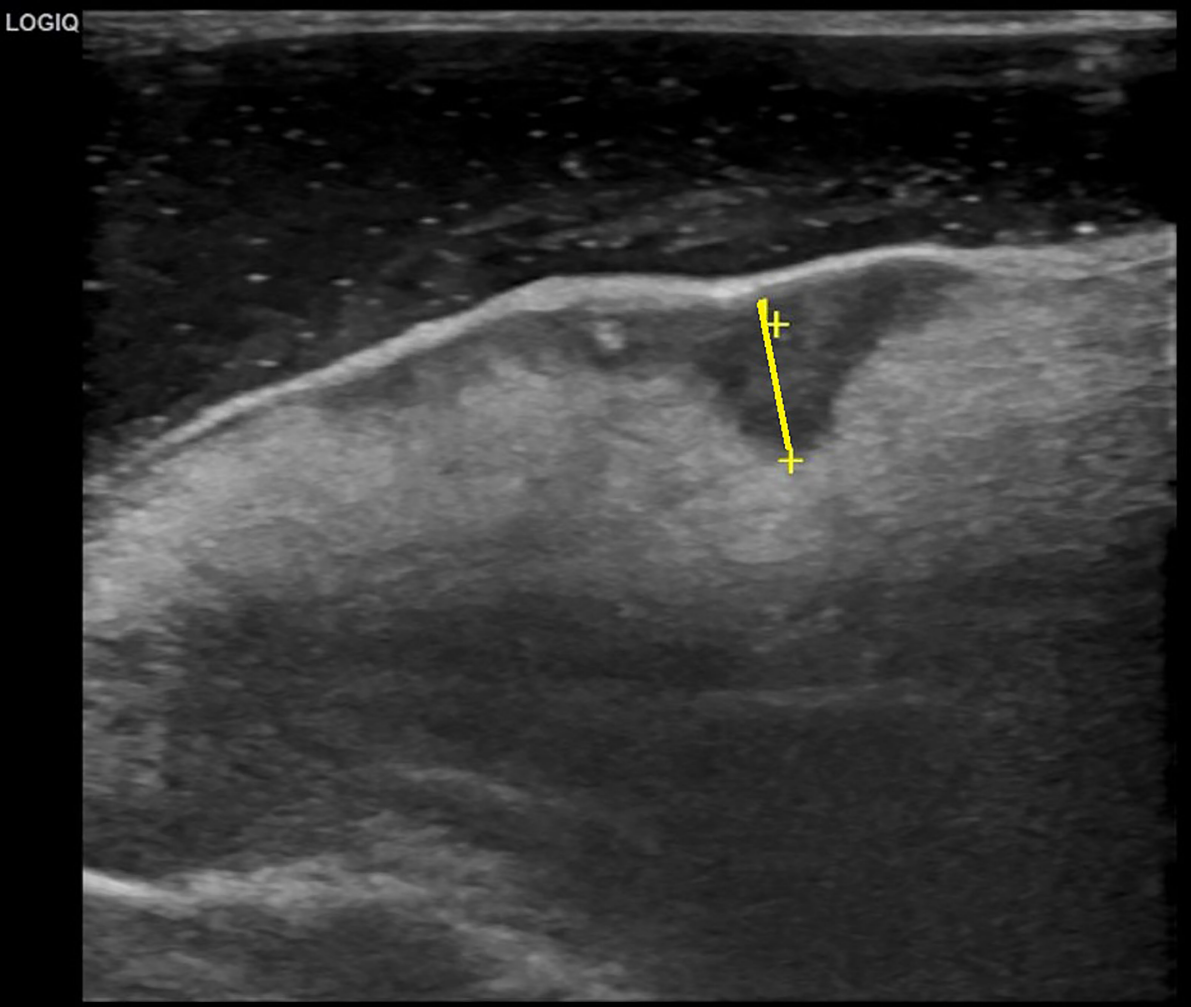
Measurement of sonographic depth of invasion (yellow line).

Sonographic DOI was defined as the vertical distance between the deepest part of the tumor and the virtual line connecting the normal mucosal basal portion adjacent to the tumor (17). Before evaluation, all patients were required to rinse the mouth. Stationary B-mode ultrasound was performed with a 10–12 MHz intracavitary probe (SonoScape, Shenzhen, China) using degassed water as the coupling agent. The tongues of the patients were lightly held with gauze, and the intraoral probes were positioned according to the longitudinal axis of the maximum diameter of the tumor. Scanning was performed with the probe in contact with the lesion, but without compression, to avoid distortion and alteration of the DOI (**Figure 1**).

### Treatment Principle

Sonographic DOI was frequently assessed for tongue SCC patients from January 2015 in our department. Resection of the primary tumor was performed with a margin of at least 1 cm. The neck management consisted of two strategies: OBS and END. END consisted of suprahyoid neck dissection (SOND) and modified radical neck dissection (MRND). SOND is referred to as a dissection of level I to III, whereas MRND is referred to as a dissection of level I to IV/V. The final neck treatment was based on the preference of the surgeon and the condition of the patient. Postoperative radiotherapy was suggested in cases with cervical nodal disease, positive margin, PNI, LVI, and extracapsular extension. Patients were followed up every three months for the first two years, every six months for the third to fourth year, and once yearly thereafter.

### Statistical Analysis

The ROC curve was used to analyze the optimal cutoff value of sonographic DOI in predicting occult metastasis. Bland–Altman and Spearman rank correlation analyses were used to compare sonographic and pathologic DOIs. The chi-square test was used to compare the clinicopathologic variables between the two DOI groups. The main study endpoints were regional control (RC) and DSS. RC time was calculated from the date of surgery to the date of the first neck cancer recurrence or the last follow-up. DSS was calculated from the date of surgery to the date of cancer-related death or the last follow-up. The Kaplan–Meier method (univariate analysis) was used to analyze the RC and DSS rates. Factors which were significant in univariate analyses were then analyzed in Cox model to find out the independent predictor for the survival. All statistical analyses were performed using SPSS 20.0, and p <0.05 was considered significant.

## Results

### Baseline Data

A total of 178 patients (135 men, 42 women) were included in the analysis; the median age was 53 (range: 28–78) years. Smokers and drinkers comprised 100 (56.2%) and 50 (28.1%) patients, respectively. Sixty-five (36.5%) patients underwent OBS for neck treatment, and 113 (63.5%) patients underwent END, with 70 (39.3%) undergoing SOND and 43 (24.2%) undergoing MRND. The mean sonographic DOI was 3.8 (range: 0.4–5.0) mm.

Postoperatively, all patients were pathologic stage T1, and clear margins were noted on histopathologic examination. Pathologic neck lymph node metastasis occurred in 12 patients (10.6%, 12/113), of whom six received SOND and six received MRND. Level I, II, III, and IV metastases were noted in 10 (5.6%), three (2.7%), three (2.7%), and one (0.9%) patient, respectively. PNI and LVI were present in 17 (9.6%) and 13 (7.3%) patients, respectively. The tumors showed good differentiation in 72 (40.4%), intermediate differentiation in 84 (47.2%), and poor differentiation in 22 (12.4%) patients. The two groups had similar distributions regarding clinical and pathologic variables (all p >0.05, **Table 1**).

**Table 1:** Comparison of clinical and pathologic variables between the observation and elective neck dissection groups.

Variables	Observation (n = 65)	Elective neck dissection (n = 113)	p
Age			
<40	8	14	
≥40	57	99	0.987
Sex			
Male	50	86	
Female	15	27	0.902
Smoking	40	60	0.274
Drinking	20	30	0.546
Sonographic DOI*			
<4.0 mm	37	63	
≥4.0 mm	28	50	0.880
PNI^&^	7	10	0.675
LVI^^^	5	8	1.000
Differentiation			
Well	24	47	
Intermediate	33	52	
Poor	8	14	0.810

*DOI, depth of invasion.

^&^PNI, perineural invasion.

^LVI, lymphovascular invasion.

### Adjuvant Treatment

Radiotherapy was performed for 30 patients, of whom six underwent radiation for the primary site, 12 underwent radiation for the primary site and ipsilateral upper neck area, and 12 underwent radiation for the primary site and ipsilateral neck area.

### ROC Curve of Sonographic DOI

**Figure 2:**
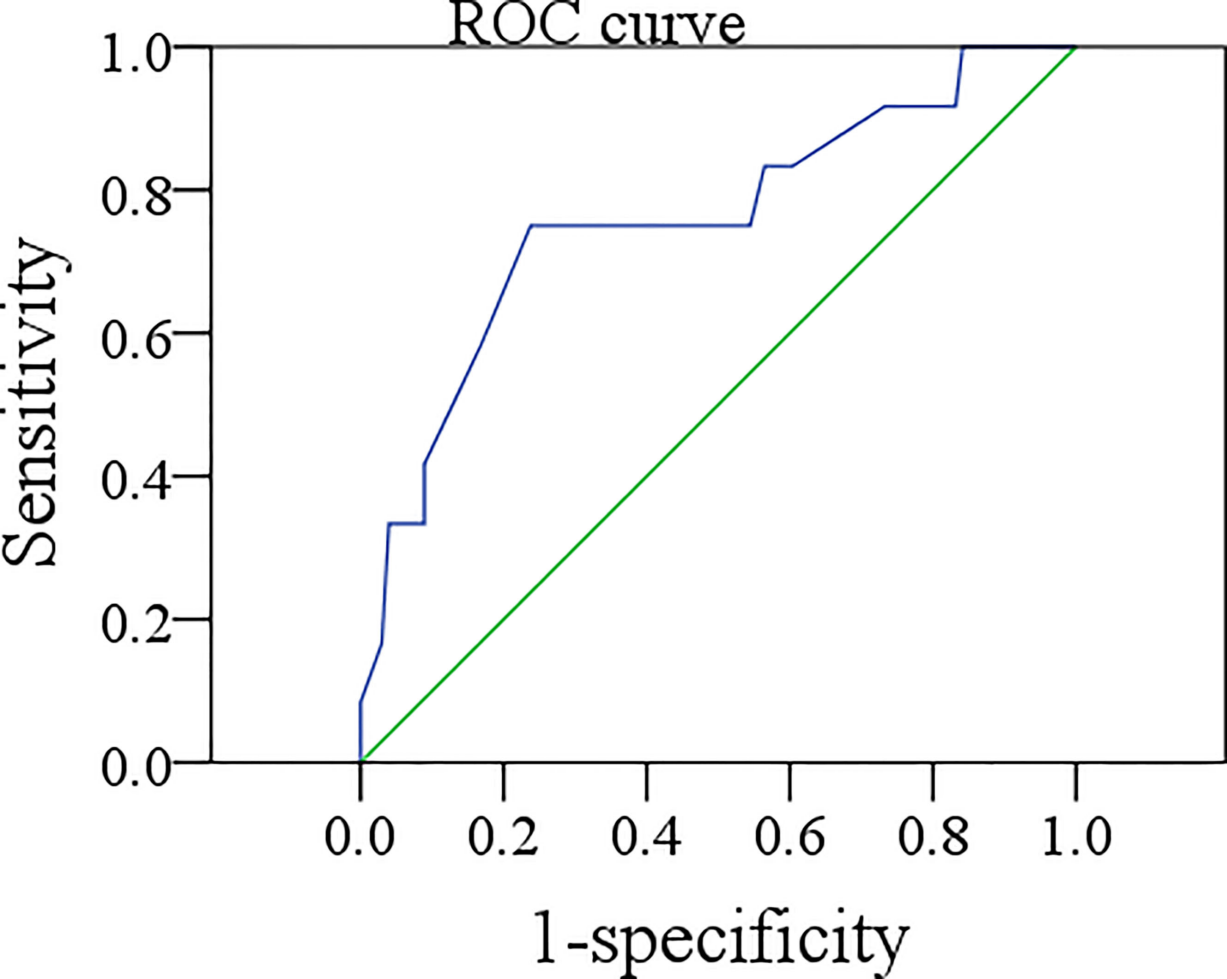
ROC curve of the sonographic depth of invasion in predicting occult metastasis.

In the END group, the mean sonographic DOI was 3.8 (range: 0.5–5.0) mm. ROC analysis indicated that the best cutoff value for sonographic DOI in predicting occult metastasis was 4.0 mm, with an AUC of 0.759 (**Figure 2**), sensitivity of 75%, and specificity of 59.4%. Eighteen percent of the 50 tumors with sonographic DOI ≥4.0 mm had occult metastases, which was significantly higher than the 4.8% of the 63 tumors with sonographic DOI <4.0 mm (p = 0.031).

### Association Between Sonographic DOI and Pathologic DOI

**Figure 3:**
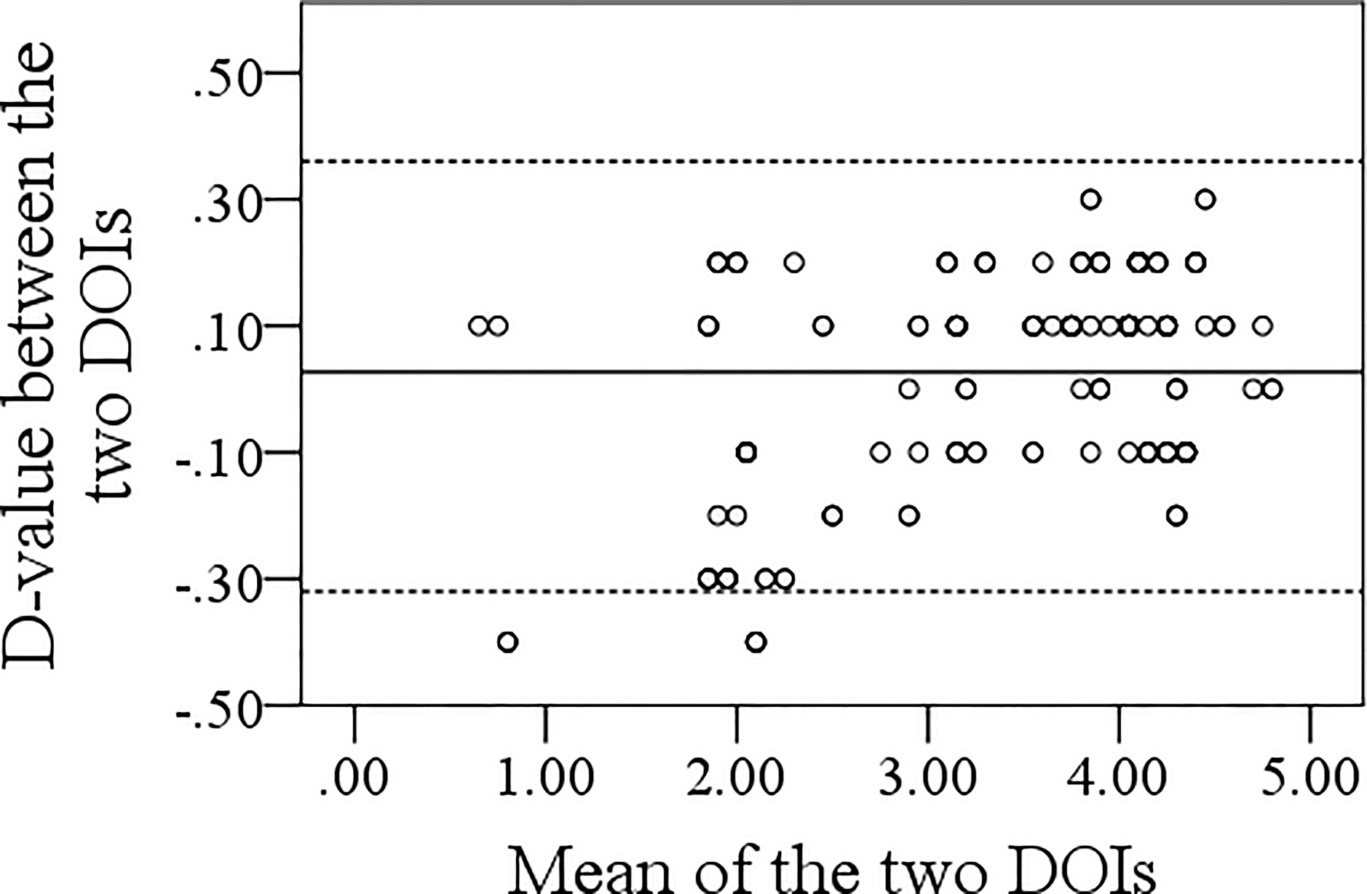
Bland–Altman analysis of the association between sonographic and pathologic depth of invasion.

The mean pathologic DOI was 3.7 (range: 0.3–4.8) mm. Spearman analysis of the relationship between sonographic and pathologic DOI yielded a correlation coefficient of 0.974 (p <0.001). Bland–Altman analysis indicated that the sonographic DOI corresponded to the pathologic DOI (**Figure 3**).

### Neck Recurrence Pattern

In tumors with sonographic DOI <4.0 mm, neck recurrence occurred in six and five patients in the OBS and END groups, respectively. In the OBS group, the most common recurrent site was level I; contralateral level II and III recurrence occurred in one patient each. In the END group, the most common recurrent site was level I, while contralateral level II recurrence occurred in one patient. The two groups had a similar recurrence pattern (**Table 2**).

**Table 2:** Neck recurrence pattern in the observation and elective neck dissection groups stratified by different ultrasound derived depth of invasion (DOI).

Level	Observation (n = 18)				Elective neck dissection (n = 14)			
	Ultrasound derived DOI ＜4.0 mm		Ultrasound derived DOI≥ 4.0 mm		Ultrasound derived DOI ＜4.0 mm		Ultrasound derived DOI ≥4.0 mm	
	Ipsilateral	Contralateral	Ipsilateral	Contralateral	Ipsilateral	Contralateral	Ipsilateral	Contralateral
I	4		4		3		5	
II	2	2	4	2	2	1	3	1
III	2	1	4	2	1		2	2
IV	1		2	2	1		1	
V			2					

In tumors with sonographic DOI ≥4.0 mm, neck recurrence occurred in 12 and nine patients in the OBS and END groups, respectively. In the OBS group, level V recurrence occurred in two patients, while level I, II, and III recurrence occurred in one patient each. In the END group, the most common recurrent site was level I, while contralateral level II and III recurrence occurred in one and two patients, respectively. The recurrence pattern in the OBS group was more complex (**Table 2**).

### RC and DSS

**Figure 4:**
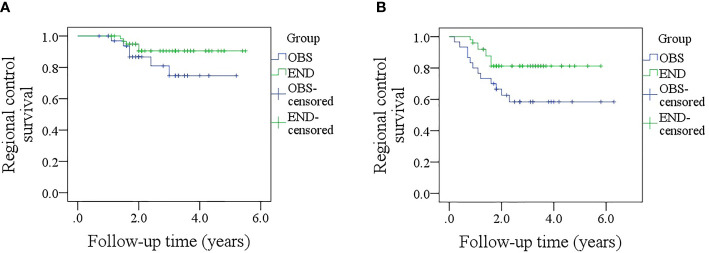
**(A)** Comparison of regional control rates between the elective neck dissection and observation groups in patients with a sonographic depth of invasion <4.0 mm (p = 0.139); **(B)** Comparison of regional control survival between the elective neck dissection and observation groups in patients with a sonographic depth of invasion ≥4.0 mm (p = 0.031).

After a median follow-up of 2.8 (range: 0.3–6.3) years, in patients with sonographic DOI <4.0 mm, the 5-year RC rates were 73 and 89% in the OBS and END groups, respectively; the difference was not significant (**Figure 4A**, p = 0.139). In patients with sonographic DOI ≥4.0 mm, the 5-year RC rates were 57 and 80% in the OBS and END groups, respectively, and the difference was significant (**Figure 4B**, p = 0.031). Further, Cox model analysis confirmed that neck dissection was an independent factor for improving RC (**Table 3**).

**Table 3:** Univariate and cox model analyses of regional control survival in patients with ultrasound derived DOI ≥4.0 mm.

Variable	Univariate analysis	Cox model	
	Log-rank	p	HR [95% CI]
Age (<40 vs ≥40)	0.356		
Sex	0.667		
Smoking	0.214		
Drinking	0.772		
DOI of ultrasound			
<4.0 mm			
≥4.0 mm	0.031	0.011	2.565 [1.223–4.787]
Positive lymph node	<0.001	<0.001	3.227 [1.835–7.218]
PNI	0.034	0.103	2.643 [0.785–9.116]
LVI	0.117	0.345	
Differentiation			
Well			
Intermediate		0.056	2.082 [0.946–4.897]
Poor	<0.001	<0.001	3.776 [2.001–6.438]

**Figure 5:**
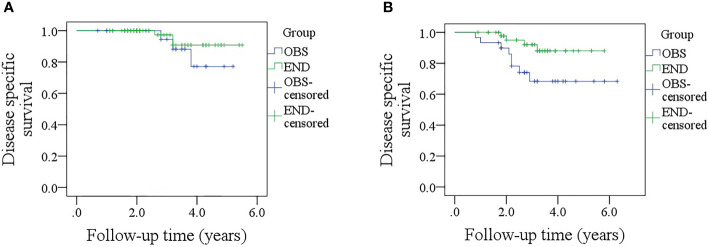
**(A)** Comparison of disease-specific survival between the elective neck dissection and observation groups in patients with a sonographic depth of invasion <4.0 mm (p = 0.381); **(B)** Comparison of disease-specific between the elective neck dissection and observation groups in patients with a sonographic depth of invasion ≥4.0 mm (p = 0.033).

In patients with sonographic DOI <4.0 mm, the 5-year DSS rates were 79 and 89% in the OBS and END groups, respectively, and the difference was not significant (**Figure 5A**, p = 0.381). In patients with sonographic DOI ≥4.0 mm, the 5-year DSS rates were 67 and 86% in the OBS and END groups, respectively, and the difference was significant (**Figure 5B**, p = 0.033). Further, Cox model analysis confirmed that neck dissection was an independent factor for improving DSS (**Table 4**).

**Table 4:** Univariate and cox model analyses of disease specific survival in patients with ultrasound derived DOI ≥4.0 mm.

Variable	Univariate analysis	Cox model	
	Log-rank	p	HR [95% CI]
Age (<40 vs ≥40)	0.221		
Sex	0.436		
Smoking	0.178		
Drinking	0.383		
DOI of ultrasound			
<4.0 mm			
≥4.0 mm	0.033	0.009	2.667 [1.567–4.328]
Positive lymph node	<0.001	<0.001	3.415 [1.675–9.287]
PNI	0.026	0.176	2.007 [0.811–8.142]
LVI	0.228	0.226	
Differentiation			
Well			
Intermediate		0.026	2.432 [1.761–5.205]
Poor	<0.001	<0.001	4.036 [1.935–8.328]

## Discussion

The most important finding in this study was that the sonographic DOI corresponded with pathologic DOI. There was a significant survival difference between the OBS and END groups in patients with cT1N0 tongue SCC with sonographic DOI ≥4.0 mm but not in patients with sonographic DOI <4.0 mm. This finding provides a clear indicator for neck management; hence, END was suggested if there was a presence of sonographic DOI ≥4.0 mm.

Neck lymph node metastasis is an important feature of tongue SCC, and its prevalence differs with tumor stage; END is usually recommended when the estimated risk of lymph node metastasis exceeds 20% (23). However, current evidence suggests that the incidence of occult metastasis in cT1N0 tongue SCC varies from 5 to 10% (6), contributing to debates regarding neck management in patients. A recent high-quality study by D’Cruz et al. (8) showed that, in the results of the first 500 patients with early-stage oral SCC, END resulted in higher overall survival and DSS rates than OBS. However, de Bree et al. (24) discussed the importance of a clear definition of cN0. Questioning the reliability of investigations for this diagnosis, they argued that cN0 was not clearly defined in the Tata Memorial Centre prospective randomized trial; further, to examine the role of ultrasound, some patients with suspicious findings were included, and more importantly, the ultrasound scoring criteria were not described. It was clear that the incidence of delayed metastases and neck recurrence would have been higher if the neck status was staged only by palpation compared with staging using advanced diagnostic techniques. Similar studies reported conflicting results on the benefits of OBS vs END (3–7); thus, there is a need for a reliable preoperative predictor of lymph node metastasis.

DOI was considered for tumor staging in the newest version of the AJCC classification, and it was confirmed as the strongest predictor of lymph node metastasis (11–16), according to the NCCN guidelines (13), END was suggested if pathologic DOI >4.0 mm existed. Pathologic DOI was calculated from the basement membrane to the deepest of invasion, although it was impossible to take the same measurement method, it was important to draft an alternative preoperative indicator of pathologic DOI to create a balance between overtreatment and necessity of lymphadenectomy.

Intraoral ultrasound has gained interest since its introduction by Iro et al. for assessing the tongue and the floor of the mouth (25), and a number of researchers have analyzed the accuracy of intraoral ultrasound in evaluating the DOI of oral SCC patients. Iida et al. (26) found in 56 cases of tongue tumor that the median ultrasound DOI was 3.6 (range: 0.7–9.2) mm, and the median histologic DOI was 3.5 (range: 0–12.0 mm). Compared to histologic DOI, there was an overestimation by only 0.1 mm for ultrasound DOI, with a coefficient of 0.867. If only superficial tumors were analyzed, the compatibility between the two DOIs improved. In another study by Yoon et al. (27) consisting of 22 patients, the mean sonographic DOI and histologic DOI were 6.6 ± 3.4 and 6.4 ± 4.4 mm, respectively, and there was excellent correlation between sonographic and histologic measurement for DOI, with a Pearson correlation coefficient of 0.95 (95%CI: 0.87–0.98). Filauro et al. (28) also noted that the mean difference between sonographic DOI and histologic DOI was only 0.3 mm after analyzing the outcome of 49 patients with cT1-3 tongue SCC, and the two DOIs were significantly related. Together with our results, these findings indicate the high reliability and accuracy of DOI evaluation by ultrasound even in cT1 tumors.

The association between the necessity of END and DOI has been frequently analyzed. Nguyen et al. (29) included 70 patients with cT1N0 oral SCC, of whom 27 underwent END and 43 were observed. Regional relapse occurred in 16.3% of patients who were observed and in 3.7% patients who underwent surgery. Risk factor analysis reported that DOI ≥3.0 mm was related to a poor prognosis, and it was concluded that END should be recommended if DOI ≥3.0 mm. However, the sample size of this study was notably small, and more importantly, it analyzed all regions of the oral cavity together. It is well known that tongue SCC has a significantly different biologic behavior compared to SCC of other oral regions. Kuan et al. (30) recently conduct a review to determine the optimal cutoff DOI value for predicting regional disease for early-stage tongue SCC, and noted that patients with cT1-2N0 oral/tongue SCC with known DOI >3.0 mm should be counseled on the possible survival benefits of END with primary tumor resection. However, the review only focused on the association between regional metastasis and DOI without considering the oncologic outcome. However, compared to T2 disease, a T1 tumor has a lower possibility of occult metastasis, which necessitates a search for a corresponding DOI for each disease stage. To the best of our knowledge, this is the first study to analyze how clinical sonography affects oncologic outcomes in patients undergoing different neck management strategies. Our study indicated that END improved patient prognosis for sonographic DOI ≥4.0 mm, but there was no apparent survival benefit associated with END for sonographic DOI <4.0 mm. This finding provides a useful method to aid decision-making in clinics.

Other studies have compared END and OBS in early-stage oral SCC. In a previous study, we enrolled 175 patients with cT1N0 buccal SCC, and the 5-year locoregional control rates in the END and OBS groups were 92 and 90%, respectively, and the difference was not significant. Moreover, the two groups had comparable 5-year DSS rates. Therefore, we concluded that END did not provide any survival benefit compared to a wait-and-watch policy and could not be suggested for patients with cT1N0 buccal SCC (31). A similar viewpoint was offered by Vandenbrouck et al. (3), Fakih et al. (4), and Yuen et al. (5). However, Huang et al. (32) analyzed the outcome of 380 patients with cT1-2N0 tongue SCC and reported the 5-year overall survival and neck control rates were significantly better in the END group than in the OBS group. Their conclusion was also supported by Abu-Ghanem et al. (6), Ren et al. (7), D’Cruz et al. (8), and de Bree et al. (24). However, these studies did not present the results stratified by the clinical DOI. As DOI is the strongest predictor of occult metastasis, the significance of our study is well highlighted.

The limitations in current study must be acknowledged. First, the study was retrospective with the attendant bias. Second, our sample size was not sufficiently large, we could not analyze the effect of the END extend on the outcome; hence, future studies with a larger sample size need to be conducted.

In conclusion, sonographic DOI corresponded well with pathologic DOI, and there was a significant survival difference between the OBS and END groups in patients with cT1N0 tongue SCC with sonographic DOI ≥4.0 mm but not in patients with sonographic DOI <4.0 mm. Our findings provide a useful method to aid decision-making in the clinic setting for this patient group.

## Data Availability Statement

The original contributions presented in the study are included in the article/supplementary material. Further inquiries can be directed to the corresponding author.

## Ethics Statement

This study was approved by the Institutional Research Committee of our hospital, and all the participants signed an informed consent. The patients/participants provided their written informed consent to participate in this study.

## Author Contributions

All authors listed have made a substantial, direct, and intellectual contribution to the work and approved it for publication.

## Conflict of Interest

The authors declare that the research was conducted in the absence of any commercial or financial relationships that could be construed as a potential conflict of interest.

## Publisher’s Note

All claims expressed in this article are solely those of the authors and do not necessarily represent those of their affiliated organizations, or those of the publisher, the editors and the reviewers. Any product that may be evaluated in this article, or claim that may be made by its manufacturer, is not guaranteed or endorsed by the publisher.
